# Gender Stereotypes and Perceptions of Stranger Violence: Attributions of Blame and Motivation

**DOI:** 10.1177/0306624X221124849

**Published:** 2022-10-12

**Authors:** Amber Phillips, Melissa S. de Roos

**Affiliations:** 1 University of Roehampton, London, UK; 2 Erasmus University Rotterdam, Rotterdam, The Netherlands

**Keywords:** gender, stranger violence, intimate partner violence, violent crime, stereotypes

## Abstract

Violence is a harmful, complex and gendered act that impacts individuals and communities financially, physically, socially and psychologically. Many studies have investigated how and why gender impacts perceptions of intimate partner violence (IPV), but little research has investigated its effect on stranger violence, despite being publicly perceived as causing greater fear and harm. Th is study examines the effects of gender attitudes and stereotypes on perceptions of stranger violence, specifically attributions of blame, affective response, alleged motivations and attitudes regarding the acceptability of violence (in general). Data was collected from 265 United Kingdom (UK) adults using an online survey posted on Amazon’s Mechanical Turk (MTurk). Results indicate that like IPV, gender affects perceptions of stranger violence. Male and female perpetrators of stranger violence are perceived differently, and gender differences are evident between male and female participants. Practical implications of the findings are discussed.

## Introduction

Mindless, random and impulsive acts of brutality reported in the media fuel moral outrage, unsettle the predictability of the environment, increase social instability and lead to a disproportionate fear of victimization (Blackburn, 1993). The long-standing disparity between the perception and reality of interpersonal violence is well-documented and empirically supported. For example, despite most violent crime being committed by those we know (61%) as opposed to strangers (38%) (Office for National Statistics [ONS], 2019) many people are most afraid of victimization by strangers (Hessick, 2007; Leverentz, 2012). Moreover, violent crimes (such as rape) that are perpetrated by strangers as opposed to acquaintances, family members or a spouse are perceived as more harmful and serious (Ben-David & Schneider, 2005) despite the experience for victims being as equally psychologically damaging and severe (Koss et al., 1988).

Violent crime is a serious public concern (Coleman, 2018) with detrimental immediate and long-term physical, psychological, social and financial consequences (Cohen et al., 1994). In the UK, violent crime is characterized as the excessive and illegitimate use of force, and it incorporates a wide range of criminal offenses including assault, robbery, homicide and rape (Blackburn, 1993). Violent crime is universally accepted as a largely male phenomenon as men dominate media reports, official publications and statistics of violent crime, while women make up much smaller numbers (Zimmerman & Messner, 2010). According to the Crime Survey for England and Wales (ONS, 2019), men accounted for the highest proportion of adult perpetrators (74%) and victims (65%) of violent crime in 2018. However, the gender crime gap is shrinking as female-perpetrated violent crime is increasing (Carrington, 2013). For example, Arnull and Eagle (2009) found that in the UK violent offenses committed by female juveniles doubled between 2000 and 2005. Nevertheless, public reactions to male-perpetrated as opposed to female-perpetrated violent crime vary wildly (Feather, 1996; Hamby & Jackson, 2010).

In 1999 in Finland, Sanna Sillanpàà opened fire at five men, killing three of them and injuring a fourth, and was characterized as “sick,” “bad” and a “ruthless killer” (Berrington & Honkatukia, 2002). In 2013 Jodi Arias was found guilty of murdering her ex-partner (Scarduzio et al., 2017), despite claiming self-defense due to a “controlling, psychologically abusive relationship” (McLaughlin, 2013, para.18). Both cases attracted widespread global media interest and while illustrating severe cases of female-perpetrated violent crime, they catalyzed the controversial debate about public perceptions of male and female perpetrators of violence (Scarduzio et al., 2017). These cases emphasize how female violence is viewed more dichotomously with perpetrators portrayed as either mentally unwell, emotionally unstable or cold-hearted (Brennan & Vandenberg, 2009) or as victims of circumstance and acting in self-defense (Saunders, 1986).

Much of the research looking at perceptions of female-perpetrated violence compared to male-perpetrated violence have focused on Intimate Partner Violence (IPV). In this context, several studies suggest that the public excuse and downgrade female brutality (Rhatigan et al., 2011), and numerous studies have consistently found that people attribute less responsibility and blame to female perpetrators of IPV and view the consequences of their violent behavior as less severe compared to male-perpetrators (Capezza et al., 2021; Rhatigan et al., 2011; Sorenson & Taylor, 2005). Furthermore, male-perpetrated IPV is regarded as more blameworthy (Cook & Harris, 1995), more criminal (Ferguson & Negy, 2004), and perceived as generating greater victim fear (Russell et al., 2019) relative to female-perpetrated IPV. A breadth of evidence also suggests that male IPV is perceived as more problematic, culpable, and morally egregious compared to female IPV (Rhatigan et al., 2011). However, relatively few studies have specifically examined the cause(s) of these perceptions which may have important implications for violence prevention policy, intervention and treatment programs. Further, this research exclusively focuses on violence in the context of an intimate relationship. Comparatively little is known about perceptions of female-perpetrated violence in other contexts.

### Gender Stereotypes and Perceptions of Violence

Stereotypes shape the way individuals make sense of the world and allow the application of labels and qualities to whole groups for quicker information processing and evaluation (Cameron, 1988). Gender stereotypes reinforce cultural expectations of acceptable and unacceptable behavior for men and women (Burkette & Warchol, 2009). According to Renzetti (1999), societies construct gender in oppositional terms (i.e., what women are, men are not and vice versa). Women are often characterized as maternal, nurturing, passive, and physically and emotionally weak (Skeggs, 2001), which may explain why female perpetrators of IPV are often perceived as less guilty, frightening or capable of causing harm compared to males perpetrators (Russell et al., 2019) or as victims and acting in self-defense (Saunders, 1986). Conversely, women who exhibit violent tendencies toward an intimate partner are often portrayed as abnormal, hormonally or emotionally imbalanced, acting under the influence of alcohol or drugs, or as inherently evil (Carlyle et al., 2014). A few high-profile cases illustrate female-perpetrated violence outside the context of an intimate relationship is perceived in a similar manner. For example, serial killer Aileen Wuornos was described by the media as a “deadly rattlesnake” (Morrissey, 2003, p. 39), and depicted as a cold-hearted, cunning, manipulative “non-woman” who was motivated by greed (The Associated Press, 1992). Such characteristics are often applied to female perpetrators of violence eliciting public outrage and fear, and to explain their rebellion against stereotypical notions of femininity (Franklin, 2008; Irwin & Chesney-Lind, 2008) in order to minimize their blame and culpability. However, these alleged motivations rely principally on gender stereotypes and fail to excogitate psychologically-grounded theories and evidence, which arguably hinders the exploration and understanding of female criminality. Views like these also negatively impact women’s experiences of the criminal justice system; a system designed for men by men, and whilst this has been addressed in recent years, much more remains to be done.

Men are also stereotyped, particularly regarding their masculinity and/or status as a victim (Whitehead, 2005). Despite the fluid and dynamic nature of masculinity, one construction of male identity is hegemonic and promotes heterosexuality, the subjugation of women, and competitiveness among men (Connell & Messerschmidt, 2005). Consequently, male violence is seen as self-motivated, typically as an intentional response to infidelity, jealously or anger, utilized to intimidate, control or exert power (Sadeh et al., 2012; Saunders, 2002), or to reaffirm their masculinity (Messerschmidt, 2012). Therefore, within the context of violence, men are more likely to be perceived as the perpetrators or instigators, inflicting more severe injury and ascribed higher levels of blame and criticism relative to women (Russell et al., 2019; Scarduzio et al., 2017). Furthermore, men are often perceived as illegitimate victims (West & Zimmerman, 1987) particularly those not conforming to traditional notions of hegemonic masculinity, such as homosexual men (Allison & Klein, 2021).

### The Present Study

While substantive literature illustrates that gender stereotypes have a significant effect on people’s perceptions of violence, research to date has primarily focused on the gender dynamics of Intimate Partner Violence (IPV), or on case studies of female perpetrators that are considered extreme deviations from the norm (i.e., Aileen Wuornos, Karla Homolka, Myra Hindley). These studies rarely address female-perpetrated violence in different contexts, such as stranger violence. As a result, the impact of gender stereotypes on stranger violence, especially gendered perceptions of this offending behavior are not well understood. To address this paucity of research, this study investigates gendered perceptions of violence between strangers, specifically how attributions of blame, affective response, acceptability of violence, and alleged motivations are affected, and makes the following predictions. (1) Participants will ascribe more blame and moral outrage to male perpetrators relative to female perpetrators and (2) female violence will more likely be attributed to emotional instability, mental health and/or substance misuse issues relative to male violence. (3) We expect participants’ general views on the acceptability of violence, as well as stereotypical beliefs about gender to impact these findings. Finally, (4) we expect to find a gender difference in both stereotypical beliefs about gender (female participants more egalitarian), and acceptability of violence (greater endorsement of acceptability of violence among male participants).

## Method

### Participants

The total number of participants recruited, composed of 164 men and 94 women from the general UK adult population (*N* *=* 265; 7 participants did not disclose their gender and were subsequently removed). Participants were recruited from MTurk which is an online crowdsourcing platform, and they received a monetary compensation of £0.20 for taking part in this study. Participants’ mean age was 32.9 years (*SD* = 10.83; range = 18–67). The majority of participants (81.4%) were White, while 5.8% identified as Black or African Caribbean, 8.9% as Asian, 0.8% as Arab and 3.1% as other or Mixed Race. Approximately, 81% of participants were British, 16% were European, and 3% reported another nationality. Most participants were either single (47.3%) or married (44.6%), and were university educated (64.3% had an undergraduate degree or higher).

### Procedure

Participants completed an online survey on Qualtrics using a published link on MTurk. After clicking on a survey link, participants were informed about the purpose and general procedures of the study. Once consent to participate was obtained, participants were randomly assigned to read one of the four vignettes that depicted a fictitious incident of stranger violence and answered a series of questions concerning their perceptions of the conflict (Attributions for Violent Behavior Questionnaire). They then completed the Gender Role Attitudes Scale (GRAS) and Socio-Demographic Questionnaire. After completing the survey, participants were debriefed. For ethical reasons, participants could decline to answer any question, but were informed that they would not be paid, and their work would be rejected, if they skipped large sections of the survey. Instructions were standardized and the presentation of materials remained consistent for all participants.

### Materials

#### Vignettes

Perpetrator and victim gender were manipulated orthogonally to create four between-subject perpetrator-victim conditions: male-female, female-male, male-male and female-female. Each vignette briefly depicted an incident of stranger violence, in a public setting. Participants were equally (MM = 61, FF = 67, FM = 65, MF = 65 respectively) and randomly assigned to read one of the four scenarios. To reduce possible bias introduced by using the terms “victim” and “perpetrator,” the names Robert, George, Kate and Emma were used in the scenarios. Initial pilot testing was conducted with around 40 friends and acquaintances of the authors to ascertain the perceived authenticity of the scenarios, and responses to the vignette were comparable across its different versions. Participants were provided with a link and given the opportunity to reflect on the vignettes. Feedback suggested the vignettes were realistic, plausible and accessible.

You are returning home from work when you notice a woman (Kate) on her phone accidentally bump into a man (Robert) on the street and spill some coffee on him. Kate apologises but Robert is not pleased and yells at Kate to watch where she is going. Kate is surprised by the response and tells Robert that he should not have been standing in the middle of the pavement. You then witness Robert slap Kate across the face, knocking her coffee and phone out of her hand. Kate responds by holding her cheek and wincing in pain. Robert walks away.

– Sample vignette male perpetrator, female victim

#### Attributions for Violent Behavior Questionnaire

This seven-item measure was based on the work of Scarduzio et al. (2017) and assessed participants’ perceptions of the conflict described in the vignette, specifically, attributions of blame, moral outrage and alleged motivation, but also their views regarding the acceptability of violence and explanations for general male- and female-perpetrated violence. A range of open-ended, closed-ended and Likert-scale questions were included to facilitate the rich, detailed and exploratory expression of ideas (Cohen & Manion, 1985), but also to generate easily interpretable and comparable quantitative data (Auriat & Siniscalco, 2005). However, open-ended question responses were not analyzed as part of the present study due to the richness of data obtained from the fixed choice questions.

An initial, closed-ended question permitted participants to ascribe blame to the perpetrator, victim, both the perpetrator and victim, or no one. A closed-ended question also ascertained perpetrator motivation with provocation, losing control and acting impulsively, suffering from mental health issues, acting deliberately and purposefully, under the influence of alcohol and drugs and/or an alternative explanation (“other”) as answer options. Participants could select more than one motivation. The remaining questions were open-ended and were not further analyzed.

#### International Positive and Negative Schedule Short-Form

Participants’ affective response to the vignette was assessed using the International Positive and Negative Schedule Short-Form (I-PANAS-SF) (Thompson, 2007). This scale comprises two five-item mood subscales measuring positive affect (PA; consisting of inspired, determined, alert, attentive and active) and negative affect (NA; consisting of upset, hostile, afraid, nervous and ashamed). Respondents indicated the extent to which they would feel each of the emotions if they had witnessed the incident described in the vignette rated on a 5-point Likert scale ranging from “1” = *Extremely* to “5” = *Very little or not at all.* The I-PANAS-SF had an acceptable internal consistency of α = .79 (PA; α = .70 and NA; α = .76 respectively).

#### Acceptability of Violence Scale

Acceptability of violence was assessed by asking participants about contexts they believed violence could be justifiable. Based on reliability analyses (Cronbach alpha) all 11 items were divided into violence for selfish reasons (e.g., it is ok to use violence to get what you want) and protective reasons (e.g., it is ok to use violence to protect your family). Respondents rated the statements using a 5-point Likert scale ranging from “1” = *Totally agree* to “5” = *Totally disagree.* This scale had a good internal consistency of α = .89.

#### Gender Role Attitudes Scale

This 20-item scale (García-Cueto et al., 2015) examined participants’ attitudes toward gender roles within the family, social contexts and employment, and consisted of five subscales: family function transcendent (FFT) (e.g., “household chores should not be allocated by sex”), social function transcendent (SFT) (e.g. “people should be treated equally, regardless of their sex”), family function sexism (FFS) (e.g., “the husband is responsible for the family so the wife must obey him”), social function sexism (SFS) (e.g., “I think it is worse to see a man cry than a woman”), and employment function sexism (EFS) (e.g., “some jobs are not appropriate for women”) (García-Cueto et al., 2015).

Gender role attitudes refer to “views held by individuals regarding the roles men and women should play in society” (van der Horst, 2014, p. 106). Transcendent (egalitarian) attitudes were defined as not discriminating against a person on the basis of sex (Beere et al., 1984), while sexist attitudes were defined as showing prejudice, stereotyping or discrimination against a person based on sex (Sibley & Wilson, 2004). Participants rated the statements using a 5-point Likert scale ranging from 1 = *Totally agree* to 5 = *Totally disagree*. The GRAS had a good internal consistency of α = .89. Transcendent items were recoded so that higher scores on each subscale and the overall scale represented more transcendent/egalitarian attitudes and lower scores represented more sexist attitudes.

##### Demographic questionnaire

A demographic questionnaire assessed gender, age, highest academic level achieved, ethnicity, marital status and nationality, as research has found that perceptions of violence can vary in accordance with these social characteristics (Craven et al., 1998; Simon et al., 2001).

### Design

The study employed a between-subjects design. The independent variables were: perpetrator and victim gender, participant gender, with gender role attitudes and attributions for violent behavior serving as covariates. The dependent variables were: who is to blame for the violent incident described in the vignette, the perceived motivations of the person who aggressed in the vignette, and participants’ emotional response to the vignette as measured by the PANAS-SF.

## Results

### Gender Differences on Gender Roles and Acceptability of Violence

First, we assessed the effect of participant gender on beliefs about gender roles as measured by the GRAS. Descriptive statistics of GRAS subscales and total scale were conducted, for men and women. A series of independent sample t-tests were conducted to examine gender differences on each subscale and the total scale. Details are displayed in Table 1. Across all subscales and the total scale, female participants were significantly more likely to hold transcendent/egalitarian views than male participants.

**Table 1.** Descriptive Statistics and Independent Sample *t*-Tests of GRAS Subscales and Total Scale.

Scale	Men		Women		*t*
	*M*	*SD*	*M*	*SD*	
Social function transcendent	16.73	2.46	17.53	2.05	−2.67**
Family function transcendent	8.44	1.61	9.39	1.18	−5.00***
Family function sexism	13.89	3.20	14.77	2.25	−2.36*
Social function sexism	14.76	3.25	17.71	2.74	−7.40***
Employment function sexism	19.21	5.13	23.70	4.96	−6.84***
GRAS total	72.97	12.04	83.31	10.67	7.04***

*** *p* < .001. **p < .01. **p < .05.

In order to assess if there were circumstances under which violence was perceived as more acceptable than others, an 11-item scale was developed. A Principal Component Analysis with Oblimin rotation was conducted to assess the uniqueness of the acceptability items. An Oblimin rotation was chosen to allow for the possibility that various circumstances where violence is deemed acceptable may be correlated. Two components with Eigenvalues above 1 were extracted, and together, these components explained 74.02% of variance. The component matrix is displayed in Table 2. Loadings below .25 are omitted.

**Table 2.** Factor Loadings based on Principal Components Analysis for Acceptability of Violence.

Item	Component	
	1	2
Violence against a partner if they have cheated	.867	
Violence to prove a point	.850	
Violence to protect your family	.297	.782
Violence to get what you want	.836	
Violence to protect yourself from immediate harm		.849
Violence to obtain a sexual act	.718	−.257
Violence if someone has humiliated you	.875	
Violence in retaliation (i.e., because someone hit you first)	.476	.661
Violence if someone has annoyed you	.908	
Violence when you disagree with someone	.879	
Violence if someone has insulted you	.893	

The identified factors appear to be violence for selfish reasons and violence for protective reasons. We created a composite score for acceptability of violence for selfish reasons by summing the items indicative of selfish-motivated violence (α = .95). Similarly, we created a composite score for acceptability of violence for protective reasons by summing the items associated with moral violence (α = .77).

The second part of our hypothesis concerned gender differences in acceptability of violence. To examine this, we conducted a series of independent sample *t*-tests. A significant effect of gender on acceptability of selfish violence was found (*t* = 2.28, *p* = .02), with men more likely to find selfish violence acceptable (*M* = 11.22, *SD* = 5.82) compared with women (*M* = 9.64, *SD* = 4.24). Similarly, gender had a significant effect on the overall acceptability of violence (*t* = 2.27, *p* = .02), with men more likely to find violence overall acceptable (*M* = 21.89, *SD* = 7.19) than women (*M* = 19.89, *SD* = 5.81). No effect of gender on acceptability of protective violence was observed.

Finally, we wanted to assess associations between stereotypical beliefs about gender and acceptability of violence. To explore this relationship, Pearson correlations between GRAS subscales and acceptability of violence were conducted. Results are displayed in Table 3. Strong, negative correlations were observed between GRAS and acceptability of selfish violence and overall violence indicating participants with more sexist beliefs were more likely to find violence acceptable. Only two subscales of GRAS correlated significantly with acceptability of protective violence.

**Table 3.** Pearson Correlations between Gender Role Attitude Scale and Acceptability of Violence.

Scale	Selfish violence	Protective violence	Overall violence
Social function transcendent	−.30***	.03	−.24***
Family function transcendent	−.36***	.05	−.29***
Family function sexism	−.59***	−.09	−.53***
Social function sexism	−.58***	−.10	−.51***
Employment function sexism	−.48***	−.20**	−.49***
GRAS total	−.60***	−.13*	−.55***

***p < .001. **p < .01. **p < .05.

#### Gender stereotypes and perceptions of violence

Our main hypothesis concerned associations of blame and perceived motivations for violence for male and female perpetrators. We first examine the attribution of blame. Across all conditions of the vignette, most participants (61.2%) blamed the perpetrator, while 26.0% blamed both the perpetrator and victim, 9.3% blamed only the victim and 3.5% blamed no one. A binary logistic regression was conducted to determine if the gender of the perpetrator, victim, or participant affected who would be blamed, but no significant effects were found.

Next, we examined perceived motivations for the incidents. In this sample, 79.1% of participants believed the perpetrator lost control and acted impulsively, 16.3% believed the perpetrator was provoked, 10.9% believed the perpetrator suffered from mental health issues, 10.5% believed the perpetrator acted deliberately and purposefully, 3.1% believed the perpetrator was under the influence of alcohol or drugs, and 3.5% offered an alternative motivation (“other”).

To examine the effects of gender, gender beliefs, and acceptability of violence on perceived motivations for violence, we conducted a series of binary logistic regression analyses. For each analysis, perpetrator, victim, and participant gender were entered as categorical predictors, and the two acceptability of violence subscales and the total GRAS scale as covariates. Each motivation was entered as a dependent variable. The logistic regression model to assess whether the perpetrator was provoked was statistically significant X^2^(6) = 15.31, *p* = .02. The model explained 10.5% (Nagelkerke *R*^2^) of the variance and correctly classified 83.8% of cases. Participants were 2.43 times more likely to report that the perpetrator was provoked when the perpetrator was male as opposed to female. None of the other variables entered had a significant effect. The logistic regression model to assess whether the perpetrator lost control was statistically significant X^2^(6) = 16.50, *p* = .01. The model explained 10.3% (Nagelkerke *R*^2^) of the variance and correctly classified 79.3% of cases. Participants were 2.22 times more likely to report that the perpetrator lost control when the perpetrator was female as opposed to male. Participants who scored higher on the GRAS were more likely to say the perpetrator lost control (*β* = .04, *p* = .02). No significant models were found for the remaining motivations.

Finally, we assessed which variables affected how the participants reported they would feel after witnessing the incident described in the vignette. Composite scores for positive affect and negative affect as measured by the I-PANAS-SF were entered as outcome variables in a MANCOVA. Participant, perpetrator, and victim gender were entered as categorical predictors, and the acceptability of violence subscales and GRAS total score were entered as covariates. The multivariate tests indicated significant effects of acceptability of protective violence and perpetrator gender on reported emotions. Acceptability of protective violence had a significant effect on negative emotions reported (*F* (1, 227) = 4.55, *p* = .03), with negative emotions increasing as the participant’s score on acceptability of protective violence increased. Perpetrator gender had a significant effect on negative emotions reported (*F* (1, 227) = 8.19, *p* = .005). Participants reported greater negative emotions if the perpetrator was female (*M* = 17.68, *SD* = 6.23) than if the perpetrator was male (*M* = 16.03, *SD* = 6.39).

## Discussion

The purpose of this study was to examine how and why gender stereotypes may impact perceptions of stranger violence, using theoretically-derived suppositions and empirical evidence. First, we found support for our hypothesized gender differences in stereotypical beliefs about gender, with female participants holding significantly more transcendent/egalitarian views than male participants. Similarly, we found a gender difference in acceptability of violence, with male participants overall more likely to judge violence as acceptable, and more likely to find violence for selfish reasons acceptable, compared with women. Secondly, we found no support for our hypothesized gender differences in attribution of blame. However, when exploring perceived motivations for violence, male perpetrators were more likely to be perceived as acting due to being provoked, whereas female perpetrators were more likely to be perceived as losing control. Finally, the gender of the perpetrator and perceived motivation for violence had an effect on participant emotions regarding the vignette, with greater negative emotions reported if the perpetrator was female, and if the participant was more likely to accept violence perpetrated for protective reasons.

### Gender Stereotypes and Perceptions of Violence

Male participants were more likely to find selfishly motivated and overall violence acceptable compared with female participants, but gender did not have an effect on the acceptability of protective violence. Perhaps this is because acting violently is incongruent with the rules and social standards of hegemonic femininity which largely rejects and disapproves of violence (Edwards, 2006), while hegemonic masculinity condones violence (illegitimate means) if a man cannot achieve his goals (e.g., status or power) legitimately (e.g., career or sport performance) (Connell & Messerschmidt, 2005). Moreover, perhaps gender had no effect on the acceptability of violence for protective reasons because violence for protective reasons is more socially ambiguous and culturally acceptable (Colb, 2019). To our knowledge, this study is the first to explore acceptability of violence in this manner. The results suggest that perceptions may be an extension of an individual difference related to the circumstances under which violence is found acceptable.

Additionally, participants with lower GRAS scores (greater sexism) were more likely to accept violence for selfish reasons and overall violence. This is not unexpected given that research has found that people who endorse sexist attitudes are also more accepting of general and specific acts of violence (e.g., IPV) (Glick et al., 2002; Herrero et al., 2017). It could be argued that people with rigid and sexist gender stereotypes tend to have a moral compass which regards inequality as inevitable and justifiable, and therefore view violence as an acceptable and appropriate strategy to achieve goals. Alternatively, it could be contended that in situations perceived to threaten a person’s dignity, ego and self-esteem, violence and sexist attitudes are viewed as permissible and proportionate strategies to counteract these threats (Guerrero-Molina et al., 2020).

We were then interested in how an incident of stranger violence would be perceived by participants. First, we examined the blame attributed to the perpetrator. It was predicted that participants would ascribe more blame to male perpetrators, relative to female perpetrators. Surprisingly, no significant difference was found between blame attributed to male and female perpetrators. Previous research has reported significant consistent differences, whereby participants ascribe less blame to female perpetrators compared to male perpetrators (Ferguson & Negy, 2004; Rhatigan et al., 2011; Sorenson & Taylor, 2005), albeit in the context of IPV. This requires further investigation, but possibly this difference is related to the fact that around 1 in 5 participants believed the perpetrator was provoked. Research suggests that in cases of bi-directional aggression (i.e., provocation or confrontation) people are motivated to believe in a just-world, whereby individuals get what they deserve (Kristiansen & Giulietti, 1990; Rhatigan et al., 2011; Sorenson & Taylor, 2005) and consequently gender may become inconsequential. Indeed, stereotypes regarding IPV remain pervasive, with a male perpetrator abusing a female victim viewed as most representative of IPV (e.g., Dutton, 2012). It is likely that different (or altogether fewer) stereotypes are activated when judging a scenario describing stranger-perpetrated violence.

We then examined the perceived motivations participants ascribed to the perpetrators. As predicted, female-perpetrated violence was more than twice as likely to be attributed to loss of control than male violence, a finding consistent with research showing that people typically perceive female perpetrators as overly emotional, out of control and impulsive (Carlyle et al., 2014; Harris et al., 2012; Scarduzio et al., 2017). This depiction of female perpetrators supports the binary gender stereotype whereby men are associated with reason and rationality, while women are associated with excessive emotion (Pavco-Giaccia et al., 2019). Notably, victim gender, participant gender, and acceptability of violence scores did not have an effect on which motivation was ascribed to the perpetrator, but GRAS score did.

On the other hand, male perpetrators were more likely to be perceived as acting in response to provocation. This finding is consistent with some research which suggests that men are more likely to externalize negative affect and respond aggressively (Verona & Curtin, 2006), and are less able to regulate their behavioral responses to emotional stimuli than women (Knight et al., 2002). Alternatively, other research suggests that male perpetrators are predominantly framed as the initiators of violence due to internal causes (e.g., a biological predisposition or anger management issues) (Scarduzio et al., 2017).

Perhaps this dissimilarity is a product of the sample demographics which comprised a young, male majority. Consequently, male participants may have selected provocation as a motive in an attempt to oppose stereotypical notions associated with male-perpetrated violence. Again, perpetrator gender was the only significant predictor of whether participants ascribed this motivation to the perpetrator. Unlike with the “loss of control” motivation, GRAS scores did not have an effect on ascribing a provocation motivation to the perpetrator.

Contrary to the prediction, the association between female violence, mental health issues, and alcohol or drugs was not significant. Research has consistently shown that female offenders are often described as “mad,” “mentally abnormal” or suffering from alcohol and/or drug abuse (Berrington & Honkatukia, 2002; Carlyle et al., 2014). A possible explanation for this difference is that because the vignettes only provided a “snapshot” of the violent encounter participants had insufficient salient information to determine the mental or physical wellbeing of the perpetrator.

Finally, we examined affective responses to the vignette. It was predicted that participants would experience more negative affect in response to a male-perpetrated incident of stranger violence. Unexpectedly, participants experienced greater negative affect when the perpetrator was female as opposed to male, a finding inconsistent with research which has found that male violence is viewed more harshly than female violence mainly because men tend to be larger in size and stronger, and thus can inflict greater fear and more severe physical injury (Capezza et al., 2021; Rhatigan et al., 2011). Possible explanations for this difference are that female violence is (or is perceived to be) less frequent and atypical, and therefore people experience greater negative affect when it is encountered. Alternatively, women who behave violently defy stereotypical notions of femininity (e.g., weak, passive and nurturing) and may therefore be viewed as more deviant to separate them from “normal” and “conforming” women, and punished more severely for challenging traditional female gender norms and values (Chesney-Lind, 1986; Estrada et al., 2019).

### Limitations

Firstly, this study only utilized written vignettes containing limited detail which have centered in debates questioning their effectiveness to capture and represent social realities (Bahmani et al., 2020). Consequently, future studies should employ alternative methods, such as digital recordings to construct events, since they could shed more light on situations or the behavior of people (Kinicki et al., 1995; Loman & Larkin, 1976) such as how people react when encountering incidents of stranger violence. Secondly, the vignettes only assessed attributions of blame and moral outrage regarding a relatively minor incident of physical violence between strangers. In cases of more severe violence, including incidents involving a weapon or sexual violence, gender stereotypes may become less or more relevant. For example, scenarios involving a weapon should eradicate the significance of size or physical strength (Rhatigan et al., 2011; Sorenson & Taylor, 2005). Alternatively, in cases of sexual violence, more emphasis may be placed on gender stereotypes especially for women, specifically, sexual promiscuity or risky behavior, dressing provocatively and consuming alcohol or drugs (Bates et al., 2019). Finally, although using MTurk resulted in a more diverse sample of the UK adult population in comparison to an undergraduate student or community sample, it is still unclear and unlikely to be representative of any population as a whole given that the corporate selection criteria for acceptance onto the platform are undisclosed and some populations who may find using the computer difficult such as those with severe mental health issues, intellectual disabilities and/or the elderly (above the age of 60) are likely to be underrepresented (Chandler & Shapiro, 2016; Chandler et al., 2019).

### Theoretical and Practical Implications

This study offers several implications. Firstly, it extends findings regarding gender stereotypes related to IPV to similar discrepant perceptions of violence perpetrated in the context of stranger violence. This underlines the importance of challenging stereotypes surrounding male and female perpetrated violence in a broader context than IPV. The first stereotype is that women are “naturally” more emotional than men. Women can be emotionless, and men can be overly-emotional (Scarduzio et al., 2017). The second stereotype is that male perpetrators are intentionally violent to demonstrate their masculinity and superiority. Raising public awareness about gender stereotypes and developing new narratives around masculinity and femininity that allow men and women to be both dominant and submissive, lose control or act in self-defense, experience fear and be physically and emotionally strong and weak, may encourage men to report being victims of violence, seek help and receive treatment. It may also promote the more equal and objective treatment of women by the criminal justice system.

Interestingly, certain gender stereotypes, such as women act violently because they are “mentally unwell” or under the influence of alcohol or drugs were extraneous regarding stranger violence. As previously stated, this is where the context in which the violence is perpetrated could play a role. This study suggests that although there are some similarities in perceptions of female-perpetrated violence in the context of an intimate relationship and a stranger relationship, there are also important differences. Perhaps fewer assumptions are made about stranger-contexts, which may activate fewer stereotypical connotations than an IPV scenario. Future research should explore perceptions of female-perpetrated violence across more diverse contexts to explore the conditions under which perceptions are particularly influenced by stereotypical ideas about gender.

Lastly, this study highlights the interconnectedness of different types of violence as gender stereotypes used predominantly (and successfully) to explain IPV can prodigiously be applied to incidents of stranger violence. While the contexts in which perpetrators express or manifest violence may differ, the underlying risk factors may be the same. Therefore, continuing to employ proactive prevention strategies to stop IPV may potentially prevent other types of violence including stranger violence. Clearly, extensive further investigation is needed to better understand how different manifestations of violence may be shaped.

## Conclusions

This study addressed the limited extant psychological literature investigating how and why gender may impact attributions of blame, affective response, alleged motivations and attitudes regarding the acceptability of violence perpetrated by a stranger. A few gender stereotypes that often manifest in discussions of IPV could be extended to the context of stranger-perpetrated violence, but some important differences related to perceived motivation and participants’ emotional reactions were found. This highlights the importance of studying perceptions of stranger violence to examine the ways in which violent acts are interconnected and understood, potentially facilitating transformative policy and practice, including public awareness campaigns, prevention and intervention strategies, refined risk assessment and management tools and education programs. An enlarged foundation of knowledge in this area may also help to counteract some of the assumptions and risk factors that facilitate and perpetuate a culture in which violence is pervasive.
